# Emergent Rashba spin-orbit coupling in bulk gold with buried network of nanoscale interfaces

**DOI:** 10.1126/sciadv.adz1680

**Published:** 2025-10-10

**Authors:** Shreya Kumbhakar, Banashree Debnath, Tuhin Kumar Maji, Binita Tongbram, Shinjan Mandal, T. Phanindra Sai, T. V. Ramakrishnan, Manish Jain, H. R. Krishnamurthy, Anshu Pandey, Arindam Ghosh

**Affiliations:** ^1^ Department of Physics, Indian Institute of Science, Bangalore 560012, India.; ^2^ International Centre for Materials Science, JNCASR, Bangalore 560064, India.; ^3^ International Centre for Theoretical Sciences, Tata Institute of Fundamental Research, Bangalore 560089, India.; ^4^ Solid State and Structural Chemistry Unit, Indian Institute of Science, Bangalore 560012, India.

## Abstract

The Rashba effect, which plays a crucial role in fundamental materials physics and potential spintronics applications, has been engineered in diverse systems, including semiconductor quantum wells, oxide heterostructures, metallic surfaces, topological insulators, ferroelectrics, etc. However, generating it in systems that preserve bulk inversion symmetry (BIS), for example, in bulk metals, has not been possible so far. We demonstrate a strategy to introduce and tune Rashba spin-orbit interaction (SOI) to unprecedented magnitudes in inversion-symmetric solids by incorporating ultrasmall silver nanoparticles in bulk gold. The near-identical lattice constants of Ag and Au allow dense packing of the Ag/Au hetero-interfaces without compromising the global BIS. By varying the density of embedded nanoparticles, we generate Rashba SOI in a bulk metal with coupling strength ~15 meV∙Å, higher than any known system preserving BIS globally, and show up to ~20 times increase in the spin-orbit scattering rate. We argue that the combined effect of charge transfer at the interfaces and polaronic localization enhances the SOI.

## Teaser

Crystalline interfaces of silver-gold hybrid reveal a strong Rashba effect, offering potential for spintronic devices.

## INTRODUCTION

Materials lacking inversion symmetry exhibit Dresselhaus or Rashba spin-orbit coupling (SOC), which lifts the spin degeneracy of the bands without an external magnetic field. Bulk inversion asymmetry (BIA) in three-dimensional (3D) bulk periodic solids, such as noncentrosymmetric zinc-blende structures, leads to the Dresselhaus SOC (*1*), whereas Rashba SOC emerges from structural inversion asymmetry (SIA), same as in 2D surfaces or interfaces (*2*, *3*). Intensive efforts have been used over several decades to engineer artificial heterostructures from metals, semimetals, semiconductors, and insulators that show Rashba coupling (*3*, *4*), which is modeled by the Bychkov-Rashba Hamiltonian $H_{R}=\alpha_{R}\vec{\sigma }.\left(\vec{k}\times \hat{z}\right)$ . Here, $\vec{\sigma }$ and $\vec{k}$ are the electron’s spin and wave vector, respectively; $\alpha_{R}$ is the Rashba parameter, and $\hat{z}$ is a unit vector along the direction of SIA, i.e., perpendicular to the surface/interface. The magnitude of $\alpha_{R}$ ranges from ~4 to 6 meV∙Å in III-V semiconductor quantum wells (*5*, *6*) to ~10 to 50 meV∙Å in interfaces of complex oxides heterostructures (*7*, *8*) to ~30 to 3000 meV∙Å on metallic surfaces or interfaces (*9*–*12*). An ongoing quest over the years is the search for materials showing bulk Rashba effect, which had been mostly associated with 2D surfaces or interfaces (*4*, *13*). This was encouraged after the discovery of giant bulk Rashba splitting in polar semiconductors such as BiTeX (X = Br, Cl, or I) (*14*–*16*), GeTe, SnTe, and organometal halide perovskites (*17*). However, bulk systems showing the Rashba effect found so far lack a center of inversion globally having a noncentrosymmetric structure and hence are observed in a restricted material domain (*13*). Because of intrinsic structural symmetry, the Rashba interaction is absent in most metals, which are important for numerous spin manipulation–related applications (*18*–*21*). This poses a fundamental question of whether it is possible to induce Rashba physics in a bulk system with global inversion symmetry.

Fundamentally, the Rashba interaction derives from the electric field associated with an interfacial potential gradient that is transformed to a magnetic field, which relativistically couples to the electron’s spin. The utilization of interfaces has hence been a potential strategy to induce the Rashba effect. This has been demonstrated even on a metallic platform, where a noncentrosymmetric artificial superlattice of [Pt/W/Co]*_N_* (*N* = 10) enables Rashba physics in bulk metallic systems (*22*), with $\alpha_{R}$ as large as ~12 meV∙Å. However, not only is the $\alpha_{R}$ strictly limited by the thickness of the metallic layers, but also its scalability to the bulk limit (i.e., large *N*) is experimentally challenging and yet to be demonstrated. Consequently, engineering the Rashba effect in a truly bulk metal that also preserves the bulk inversion symmetry remains unresolved.

In this work, we fabricate a crystalline matrix of Au that encloses multiple ultrasmall Ag nanoparticles (AgNPs) of diameter ~2 nm (*23*). By performing quantum transport measurements, we show that the hybrid system exhibits a Rashba SOC with $\alpha_{R}∼15$ meV∙Å. Our experiments suggest that this effect arises from the breaking of structural inversion symmetry and the strong dipole field generated locally at the Ag/Au interfaces because of the difference in the onsite electrochemical potentials of Au and Ag, which also leads to strong effective electron-phonon coupling (EPC) (*24*) and a soft energy gap in the electronic spectrum at low energies. The Rashba coupling could be tuned over a factor of ~20 by varying the density of nanoparticles, thereby demonstrating a unique strategy to achieve tunable Rashba interaction in a bulk noble metal by embedding a network of nanoscale interfaces.

## RESULTS

### Structural characterization

**Fig. 1.**
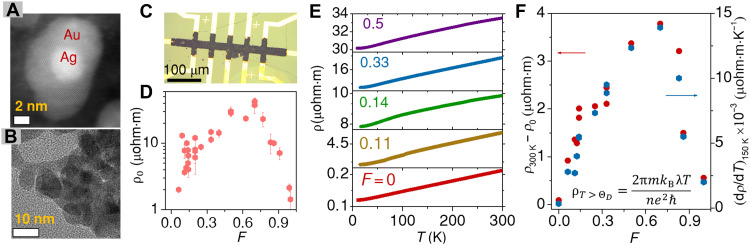
Structural and electrical characterization of Ag@Au NH films. (**A**) *z*-contrast variation in HAADF imaging of Ag@Au NH that indicates a spherical Ag nanoparticle (~2nm diameter), represented by the brighter white region, is hosted inside the Au lattice, represented by the darker gray region. (**B**) HRTEM image shows multiple AgNPs of high crystallinity embedded inside a crystalline matrix of Au. (**C**) Typical optical image of a film, represented by the darker region deposited on prepatterned Cr/Au electrodes on a glass substrate, indicated by gold-colored regions. (**D**) Variation of the residual resistivity, ρ_0_ (defined as the resistivity, ρ at the base temperature *T* ~ 6 K) with Ag-Au volume fraction, *F = V*_Ag_/(*V*_Ag_ + *V*_Au_). (**E**) *T*-dependence of resistivity, ρ for films with varying F . (**F**) *T*-dependent component of resistivity, $\rho_{300\;K}-\rho_{0}$ and the slope of ρ−T at *T* ~ 150 K are plotted in the left and right axes, respectively, for different values of *F*.

The Ag@Au nanohybrids (NHs) are synthesized with a colloidal coprecipitation process (*23*, *24*) that allows exceptional tunability in the density of the Ag/Au interfaces while retaining a global inversion symmetry owing to the near-perfect lattice matching of Ag and Au [see Materials and Methods and section S1 for the details on synthesis, which are reproduced following references (*23*, *24*)]. Figure 1A shows a typical *z*-contrast of a high-angle annular dark-field (HAADF) image of the Ag@Au NH, where the darker crystalline region of Au hosts a dispersion of the brighter region of Ag of diameter ~2 nm. Figure 1B shows the high-resolution transmission electron microscope (HRTEM) image of a section of the crystalline Au matrix embedding multiple AgNPs (*23*, *24*). It is noted from the HRTEM image that the spherical nature of the AgNPs and the Ag/Au interface is retained in the embedded structure (see section S2 for HRTEM images at different values of *F*). Our synthesis protocol offers precise tunability over the average radii of the embedded AgNPs, $r_{\text{Ag}}$ , and their volume fraction, $F=V_{\text{Ag}}/\left(V_{\text{Ag}}+V_{\text{Au}}\right)$ . For all measurements reported in this manuscript, $r_{\text{Ag}}$ is fixed at ~1 nm. Hence, the Ag-filling fraction, *F*, is also proportional to the volume density, $F/r_{\text{Ag}}$ , of the interfaces. The as-synthesized NHs are then assembled on prepatterned Cr/Au electrodes on a glass substrate with chemical “cross-linking” protocols to form a film with average thickness of ~3 μm for electrical transport measurements (*23*) (see details on film preparation in Materials and Methods and section S3). A typical optical image of a patterned film is shown in Fig. 1C, where the darker region corresponds to the Ag@Au NHs.

### Temperature-dependent resistivity measurements

We have performed four-probe resistance measurements to estimate the resistivity, ρ, of the films. Figure 1E shows the temperature (*T*) dependence of ρ for films with varying *F*. All the films are metallic down to *T* ~ 6 K, indicating the formation of a true metallic composite from the Ag@Au NH that is devoid of tunnel barrier or insulating chemical residues across the Ag/Au interfaces. Figure 1D shows the residual resistivity, ρ_0_ (ρ at temperature of ~6 K) for different Ag@Au NH films. We observe ρ_0_ to increase with increasing *F*, reaching ~30 μohm∙m at *F* ~ 0.7, which is two orders of magnitude higher compared to that of pristine Au (~0.1 μohm∙m). Such a significant enhancement arises from the buried Ag/Au interfaces that form the dominant source of scatterers and increase the resistivity almost linearly with increasing interface density, $F/r_{\text{Ag}}$ (*23*). By increasing *F* further, ρ_0_ decreases, approaching ~1 μohm∙m for a pure AgNP film (*F* = 1). Figure 1F (left axis) plots the *T*-dependent component of resistivity, $\rho_{300\;K}-\rho_{0}$ , which represents the phonon contribution to resistivity with *F*. To understand if the strong enhancement in $\rho_{300\;K}-\rho_{0}$ is due to an apparent enhancement in the EPC at intermediate values of *F* (*24*–*26*), we simultaneously show the derivative of ρ(T) with *T* (taken at $T∼\Theta_{D}=150K$ , where $\Theta_{D}$ is the Debye temperature) on the right axis of Fig. 1F. Since $\rho \approx 2\pi {\mathrm{mk}}_{B}\lambda T/\hbar {\mathrm{ne}}^{2}$ , where *n* and *m* are the electron’s number density and mass, respectively, and λ is the EPC constant (*27*), the slope of the ρ−T curve directly corresponds to λ. The correspondence between $\rho_{300\;K}-\rho_{0}$ and dρ/dT suggests strong effective EPC, enhanced by nearly ~100 times compared to that in bulk Ag or Au. Such an increase in EPC was recently suggested to arise from the charge transfer across Ag/Au interfaces (*24*, *25*). The impact of the interfaces and large EPC on the spin and spin-orbit–related processes can be nontrivial, especially due to the breaking of structural inversion symmetry at the interfaces.

### Magnetotransport measurements

**Fig. 2.**
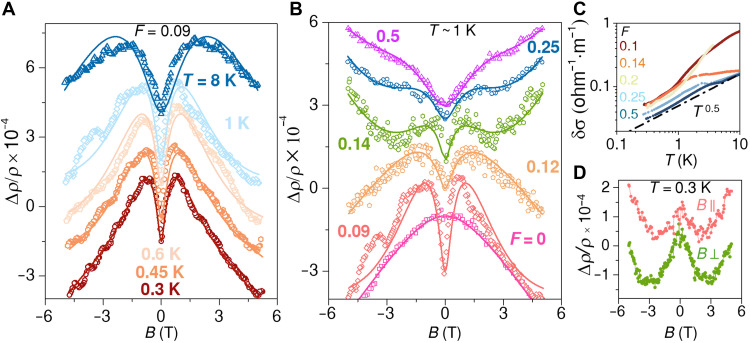
Quantum transport. (**A**) MR, represented by the relative change in resistivity  $\left\{\;\left[\rho \left(B\right)-\rho (0)\right]/\rho =\Delta \rho /\rho \right\}\;$  with magnetic field (*B*), in a perpendicular magnetic field is measured for a film with Ag/Au interface density, *F* = 0.09 at the various temperatures indicated. (**B**) MR at *T* ~ 1 K is shown for different films with values of *F* varying from 0 to 0.5. *F* = 0 represents a pure Au nanoparticle film. All the data in (A) and (B) have been shifted vertically for visual clarity. Solid lines in (A) and (B) represent fits to the data using conductivity corrections from quantum interference and EEI effects (Eq. 1). (**C**) Low-*T* transport for different values of *F* are shown. This is quantified by the correction in conductivity, $\delta \sigma =-\delta \rho /\rho^{2}$ , which is the change in conductivity ( σ=1/ρ ) with respect to *T*, from its value at *T* = 0 K ( $\sigma_{0}$ ), given by $\delta \sigma =\sigma -\sigma_{0}$ . δρ indicates the change in ρ with respect to *T*. $\sigma_{0}$ is estimated by extrapolation of the data to *T* = 0. The dashed-dotted line represents power-law behavior of δσ with *T* following ~*T*^0.5^ behavior. (**D**) MR, measured at *T* = 0.3 K, is shown for a film with *F* = 0.14 in magnetic fields applied parallel and perpendicular to the film.

To probe the SOC, we have performed magnetotransport measurements down to *T* ~ 0.3 K, with which we study the phase-coherent processes and the spin-orbit scattering (see section S4 for details on the measurement). The magnetoresistance (MR) for a film, captured by Δρ/ρ , with *F* = 0.09 is shown in Fig. 2A at *T* varying within 0.3 to 8 K. At all *T*, we observe the MR increases with magnetic fields (*B*) at lower *B* and then decreases at higher values of *B*. These represent the well-studied weak antilocalization (WAL) and weak localization (WL) behaviors observed in disordered conductors at low temperatures due to quantum interference effects (*28*–*32*) (see section S5 and S6 for results on other values of *F*). The probability of the interference depends on the phase-breaking timescale, $\tau_{\phi }$ . The nature of localization, on the other hand, i.e., WL or WAL, is determined by the spin-orbit scattering time, $\tau_{\text{soc}}$ , which introduces a relative phase in the electronic wavefunctions. Specifically, positive MR or WAL emerges from higher SO scattering, i.e., $\tau_{\text{soc}}<\tau_{\phi }$ and vice versa. We observe from Fig. 2A that the WL component decreases with increasing *T*, whereas the WAL component is almost constant. While the former behavior of MR indicates decreasing $\tau_{\phi }$ with increasing *T*, which is generally observed across different systems, the latter dependence shows an unusual enhancement in the SOC with increasing *T*. Figure 2B shows the MR for films with different values of *F* at *T* ~ 1 K. For the pure Au nanoparticle (Au NP) film (*F* = 0), we observe negative MR throughout the entire *B* range, indicating WL. With increasing *F*, we find a distinct emergence of positive MR at low *B*, suggesting WAL and, hence, enhanced SOC strength. Since Au, being heavier than Ag, has a higher intrinsic SOC (*33*), this increase of SOC with the incorporation of Ag in the hybrid system points toward an unconventional mechanism of spin-orbit scattering. We also observed another upturn in MR at higher magnetic fields for F≳0.12 , which we attribute to the effects of the electron-electron interaction (EEI) (*34*, *35*). The proportionality of δσ at low *T* with $T^{1/2}$ , shown in Fig. 2C also suggests strong EEI with increasing *F* (*34*, *36*).

To quantitatively estimate $\tau_{\phi }$ and $\tau_{\text{soc}}$ , we have fitted the quantum transport data with additive conductivity corrections from WL/WAL and EEI effects (*37*, *38*). Since conductivity correction from EEI essentially arises from the Zeeman splitting of the spin levels, we have considered the Maekawa-Fukuyama form of WL/WAL (*31*). Furthermore, we have considered a 3D model for both effects (*32*, *34*, *35*) because the MR in parallel and perpendicular fields are essentially the same, showing isotropic MR, as shown in Fig. 2D. We fit the measured quantum correction to conductivity as follows

$$\frac{\Delta \rho }{\rho^{2}}=\frac{\Delta \rho }{\rho^{2}}{\left|{}_{\text{QI}},+,\frac{\Delta \rho }{\rho^{2}}\right|}_{\text{EEI}}$$ (1)

Here, ${\left.\frac{\Delta \rho }{\rho^{2}}\right|}_{\text{QI}}$ and ${\left.\frac{\Delta \rho }{\rho^{2}}\right|}_{\text{EEI}}$ are the conductivity corrections from quantum interference and EEI effects, respectively. The analytical forms of the fit equation can be found in Materials and Methods section. It is to be noted that the quantum interefernce effects, i.e., WL/WAL, and the EEI effects differ in *T* and *B* dependences. While quantum interference gets suppressed with increasing *T* (Fig. 2A and section S5) as the dephasing rate of the electron increases, the magnetic field–dependent component of EEI enhances with *T* and always exhibits positive MR. Fits to the MR data using Eq. 1 with four fitting parameters $B_{\phi },B_{\text{soc}},g$ , and $F_{\sigma }$ are shown by the solid lines in Fig. 2 (A and B), where $B_{\phi }=\hbar /4\mathrm{eD}\tau_{\phi }$ and $B_{\text{soc}}=\hbar /4\mathrm{eD}\tau_{\text{soc}}$ are the scales of phase-breaking magnetic field and spin-orbit magnetic field, respectively, $F_{\sigma }$ is the average Coulomb interaction over the Fermi surface, and *g* is the Lande *g* factor (see sections S5 and S6 for details on the fits and fitting parameters at all values of *F* and *T*). Diffusivity, $D={\mathrm{mv}}_{F}^{2}/3{\mathrm{ne}}^{2}\rho$ , that is dependent on *F* has been estimated from the measured ρ, and constant number density, *n* = 10^28^ m^−3^, electronic mass, *m* = 10^−30^ kg, and Fermi velocity of Au, *v_F_* = 1.4 × 10^6^ m∙s^−1^ (see section S4 for Hall measurements showing the estimation of *n*).

**Fig. 3.**
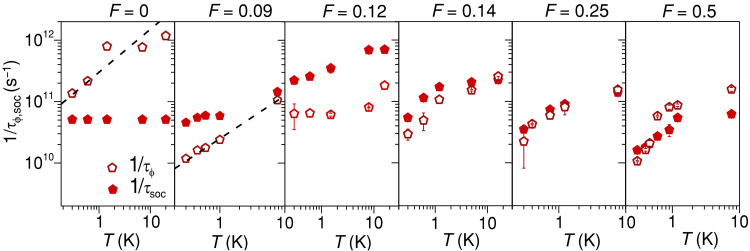
Phase-breaking and spin-orbit interaction timescales. *T* dependences of phase-breaking rate ( $1/{\text{τ}}_{\phi }$ ) and spin-orbit interaction rate ( $1/\tau_{\text{soc}}$ ) are shown for varying values of *F*. $\tau_{\text{soc}}$ and $\tau_{\phi }$ are estimated from the fits to the quantum transport data, shown in Fig. 2 (A and B) using Eq. 1. The black dashed lines in the first two panels for *F* = 0, 0.09 represent *T*^0.7^ dependence of $1/\tau_{\phi }$.

Figure 3 shows the *T* dependence of $\tau_{\phi }$ and $\tau_{\text{soc}}$ for different films with *F* ranging within 0 to 0.5. The *T* dependence of $1/\tau_{\phi }$ follows $∼T^{-p}$ behavior with p∼0.7−1 for lower Ag filling ( F≲0.09 ), indicating that EEI likely dominates the phase-breaking mechanism (*34*). With increasing *F*, the *T* dependence of $1/\tau_{\phi }$ deviates from a pure power law, becoming the weakest for *F* ~ 0.12 to 0.14, which suggests strong modification of the phase-breaking processes (see section S6 for results at other values of *F*). Secondly, the SO scattering rate, $1/\tau_{\text{soc}}$ , first increases with increasing *F* as compared to that of pristine Au until *F* ~ 0.12, after which it decreases again. We find this behavior at all temperatures, indicating it to be related to the nanostructuring of Au. Last, we find an emerging *T* dependence in $1/\tau_{\text{soc}}$ at larger *F*, which again points toward an unconventional origin of SOC in the system, likely due to the presence of interfaces.

**Fig. 4.**
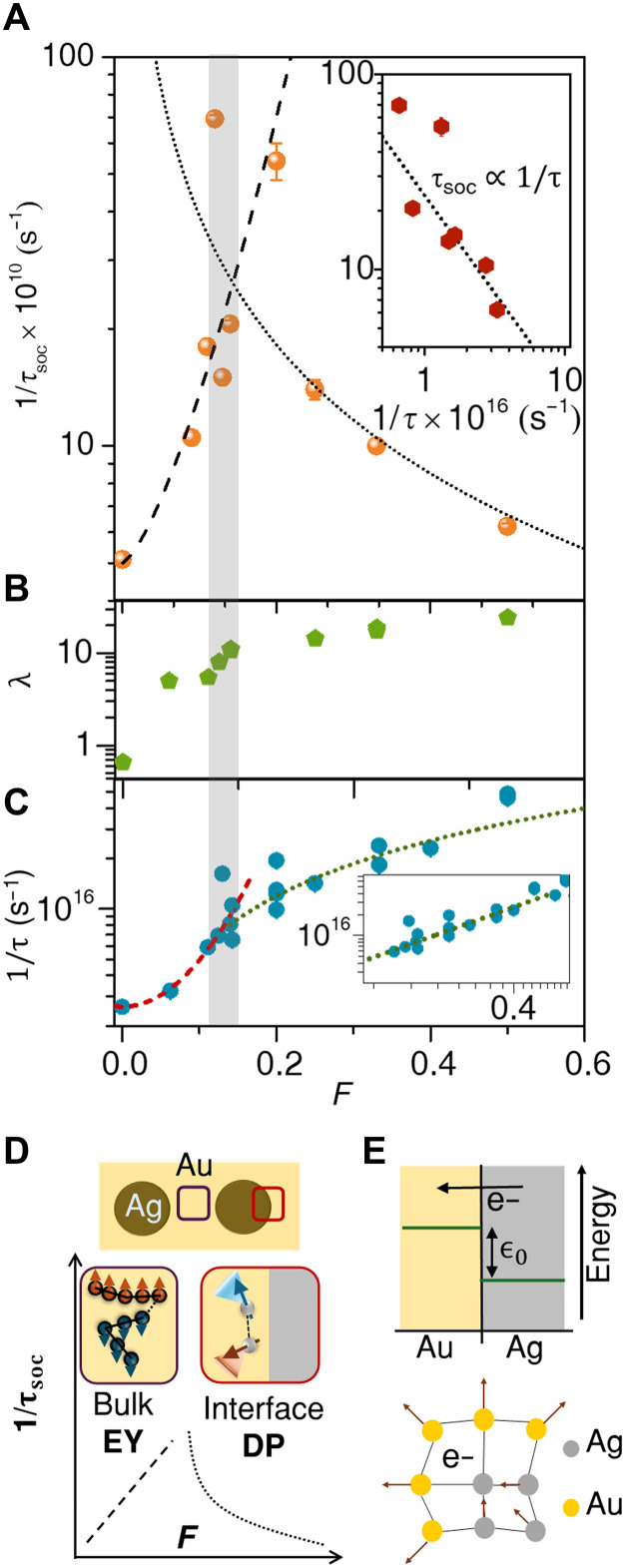
Rashba spin-orbit coupling. (**A**) Top panel shows the variation of the spin-orbit scattering rate ( $1/\tau_{\text{soc}}$ ) with *F* at *T* ~ 8 K. (**B**) EPC constant (λ), estimated from ${\left[\mathrm{d\rho}/dT\right]}_{150\;K}$ (Fig. 1F) at different values of *F* using the expression $\rho_{T>\Theta_{D}}=2\pi \lambda {\mathrm{mk}}_{B}T/{\mathrm{ne}}^{2}\hbar$ . Panel (**C**) plots the electron scattering rate ( 1/τ ), estimated from the residual resistivity, ρ_0_ (Fig. 1D) using the Drude expression of resistivity. The dashed and dotted lines in (A) represent exponential and linear dependences on *F*, respectively. The linear dependence of 1/τ on *F* is further illustrated in the inset of (C). The dashed and dotted lines in (A) represent $1/\tau_{\text{soc}}\propto 1/\tau$ and $1/\tau_{\text{soc}}\propto \tau$ , respectively, with the expressions derived from the extrapolations of 1/τ versus *F* in (C). The inset in (A) shows the proportionality of $1/\tau_{\text{soc}}$ with τ for F≳0.11 . $\alpha_{R}\approx 15$ meV∙Å is estimated from the slope of the dotted line using the expression of DP spin relaxation, $\tau_{\text{soc}}=3\hbar^{4}/8\alpha_{R}^{2}m^{2}v_{F}^{2}\tau$ . (**D**) Different mechanisms of spin relaxation are illustrated. At lower values of *F*, the EY mechanism originating from disorder potential in the bulk (purple box) dominates, whereas the DP mechanism from Rashba coupling at the interface (red box) is significant at higher values of *F*. (**E**) Top: Schematic of the electrochemical potential of electrons at the Ag and Au sites across the Ag@Au NH is shown, $\epsilon_{0}$ being the potential difference between them. Electrons transfer from a higher onsite potential at Ag to a lower potential in Au. Bottom: The polaronic trapping of electrons at the Ag/Au interface is depicted.

Figure 4A shows the *F*-dependence of $1/\tau_{\text{soc}}$ (for *T* ~ 8 K), where the nonmonotonicity of $1/\tau_{\text{soc}}$ in *F* is evident. $1/\tau_{\text{soc}}$ first increases with increasing *F*, reaching ~7 × 10^11^ s^−1^ at *F* = 0.12, which is more than an order of magnitude larger compared to that of pure Au NP film ( $1/\tau_{\text{soc}}∼5\times 10^{10}$ s^−1^). Further increase in *F* causes $1/\tau_{\text{soc}}$ to decrease, approaching ~5.5 × 10^10^ s^−1^ at *F* = 0.5. Intriguingly, we find that both λ, estimated from the slope of ρ−T in Fig. 1F, and 1/τ (calculated from the residual resistivity in Fig. 1D), undergo a change in their respective behavior at very similar values of *F*. As shown in Fig. 4B, λ increases sharply from ~0.6 at *F* = 0 to ~10 at *F* = 0.14, after which the increase in λ becomes weaker. Similarly, 1/τ also increases in a super-linear manner with *F* until *F* ~ 0.13 (red dashed line), after which it becomes almost linear (green dotted line, also in the double logarithmic plot in the inset of Fig. 4C).

Because the linear increase in 1/τ with *F* has been quantitatively shown to correspond to interface-dominated scattering (*23*), the nonmonotonic *F* dependence of $1/\tau_{\text{soc}}$ seems to represent a crossover in the nature of transport at *F* ~ 0.13, from bulk disorder dominated to that determined by the buried Ag/Au interfaces. Spin relaxation mechanisms are widely classified into two types (see the schematic of Fig. 4D). The first is the Elliot-Yafet (EY) mechanism (*39*, *40*), where the conduction electron spin interacts with its motion in the electric field of the host lattice described by the periodic potential. Here, spins of the conduction electrons relax/change due to momentum-scattering events that give rise to a spin-orbit scattering rate directly proportional to the momentum-scattering rate ( $\tau_{\text{soc}}\propto \tau$ ). The other is the Dyakonov-Perel (DP) mechanism (*41*, *42*), where inversion symmetry breaking from Rashba coupling leads to a momentum-dependent effective magnetic field, which causes spin precession and resultant dephasing. Momentum scattering, in this case, causes rapid fluctuations in the internal magnetic field and disruption of the spin dephasing. Thus, $\tau_{\text{soc}}$ induced by a Rashba interaction becomes longer as the electron scattering time, τ, becomes shorter ( $\tau_{\text{soc}}\propto 1/\tau$ ). The inset of Fig. 4A shows the dependence of $1/\tau_{\text{soc}}$ on 1/τ , where 1/τ is calculated from the magnitude of $\rho_{0}$ , consistent with the fitting of the MR data, for films with *F* > 0.12. The inverse relation (dotted line) indicates the DP-type spin relaxation, which is consistent with the possibility of structural symmetry breaking at the Ag/Au interfaces. The dotted line in Fig. 4A represents the same behavior by mapping 1/τ onto *F* (green dotted lines in Fig. 4C and inset). Quantitatively, one expects $\tau_{\text{soc}}=3\hbar^{4}/8\alpha_{R}^{2}m^{2}v_{F}^{2}\tau$ in three dimensions, which yields $\alpha_{R}\approx 15$ meV∙Å (dotted line in the inset of Fig. 4A). This is in close agreement with the Rashba interaction observed and computed at the planar interfaces created by depositing (few-layer) Ag on Au(111) substrate (*43*). Last, the dashed line describing the sharp increase in $1/\tau_{\text{soc}}$ at low *F* in Fig. 4A is representative of $\tau_{\text{soc}}\propto \tau$ , i.e., the EY mechanism, obtained by empirically fitting the 1/τ data to *F* at low values ( F≲0.12 , red dashed line in Fig. 4C). Figure 4D depicts the two regimes of spin-relaxation observed at different regimes of *F*. Figure 4E illustrates two key physical phenomena that could be intimately linked to the breaking of structural inversion symmetry and origin of the Rashba coupling. The top panel shows the charge transfer at the Ag/Au interface, driven by the difference of onsite electrochemical potential ( $\epsilon_{0}$ ) (a theoretical estimation of the charge occupancy is shown in the section S7), and bottom panel demonstrates possible polaronic distortion at the interface, linked to the giant EPC reported in these structures (*24*).

Detailed studies have shown that large Rashba splitting is observed for surface states of noble metals such as Au, Ag, and Cu (*9*, *10*, *44*–*50*) that are truly localized at the surface, and their asymmetric nature across the surface crucially determines the magnitude of the Rashba effect. This can be understood in a simple way by relating the gradient of the wave function to the electric field close to the atomic core, which leads to the momentum-dependent magnetic field $∼\left(\vec{k}\times \;\hat{z}\right)$ (*47*–*50*). The confinement of the electron gas close to the interface has been found to enhance Rashba splitting in oxide and semiconductor heterostructures as well (*51*). Hence, the dominance of the Rashba effect with increasing interface density, *F* in the Ag@Au hybrid structure, could accompany increasing localization of electronic wavefunctions close to the interface with higher *F*.

### Tunneling measurements

**Fig. 5.**
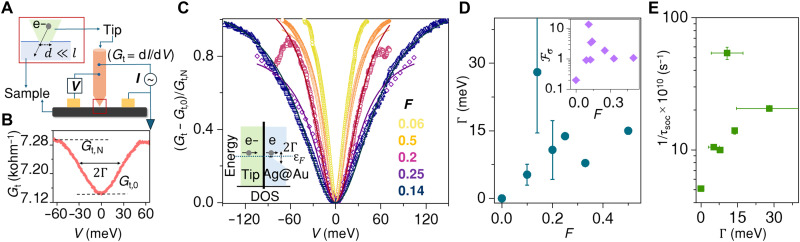
Tunneling measurements. (**A**) Schematic of the experimental setup. The tip is biased with a voltage (*V*), while the sample is grounded, thereby driving a current (*I*) across the tip-sample nanocontact of dimension *d* and tunneling conductance, $G_{t}=dI/dV$ . (**B**) Typical $G_{t}$ for a films with *F* = 0.14, measured at *T* ~ 8 K. (**C**) $G_{t}$ is shown for different films with varying *F*, measured at *T* ~ 8 K. $G_{t}$ is normalized such that the minimum value at zero bias, $G_{t,0}$ is 0, and the value saturating at larger bias, $G_{t,N}$ is 1. Solid lines are Lorentzian fits to the spectra with fitting parameter, Γ representing the width of soft gap in the electronic DOS, illustrated schematically in the inset. (**D**) Variation of Γ with *F* is shown. Inset: $F_{\sigma }$ , representing the Coulomb screening parameter, estimated from quantum transport measurements. (**E**) Variation of $1/\tau_{\text{soc}}$ at *T* ~ 8 K with Γ measured for different films with varying *F*.

We have probed the electronic density of states (DOS) by performing tunneling measurements on the films (*24*, *52*) to obtain evidence of this localization and its connection with the SOC. The experimental schematic is illustrated in Fig. 5A. A sharp metallic tip is brought in contact with the sample to measure the differential conductance ( $G_{t}=dI/dV$ ) by passing a current, *I*, through the tip at a particular bias, *V*. The dimension of the tip-sample contact was kept low for conductance to probe the tunneling DOS. Figure 5B shows the $G_{t}$ measured for a typical film of *F* = 0.14 while varying *V* within ±60 meV at *T* ~ 8 K. We observe a conductance minimum ( $G_{t,0}$ ) at zero bias, which increases and saturates at larger bias, denoted by $G_{t,N}$ . The full-width half maximum of the dip in conductance, represented by Γ is ≈30 meV. Figure 5C shows the normalized tunelling spectra at *T* ~ 8 K measured for varying values of *F*. From the homogeneity of the dip across the film and its *T*-dependence (see section S8), we exclude possibilities of disorder/impurity-mediated tunelling for the conductance minima and attribute this to opening of a soft gap, of width Γ , in the electronic DOS of the film, as represented schematically in the inset of Fig. 5C. To extract Γ precisely, we have fitted the spectra with a Lorentzian curve. Fig. 5D shows the estimation of Γ at different values of *F*. We observe Γ increases with increasing *F*, reaching a maximum of ~30 meV close to *F* ~ 0.14, after which it decreases again. The inset shows that the Coulomb screening parameter, $F_{\sigma }$ , estimated from quantum transport measurements, reaches a pronounced maximum close to *F* ~ 0.14, where Γ is also the highest. This further suggests that there is a depletion of itinerant electrons at the Fermi level, which results in a reduction of the Coulomb screening and enhanced $F_{\sigma }$.

The polaronic localization of electrons close to the Ag/Au interfaces, such as in small polarons, can enhance the wavefunction asymmetry, thus making Rashba coupling the dominant source of spin relaxation. Using both quantum transport and tunneling measurements, we observe $\tau_{\text{soc}}$ and Γ to peak around *F* ~ 0.14, implying the highest probability of localization and resulting interface-dominated electrical transport and inversion symmetry breaking. This is quantitatively captured by the direct correspondence between $1/\tau_{\text{soc}}$ and Γ in Fig. 5E. It is possible that the *T* dependence of $1/\tau_{\text{soc}}$ is also related to the temperature-driven dynamics of polarons, although further understanding is required.

## DISCUSSION

To summarize, we have demonstrated a metallic system where a Rashba SOC can be induced by a network of nanoscale interfaces. We show the coupling strength is tunable over an order of magnitude by varying the interface density (*F*), reaching a maximum value of ~15 meV∙Å. We report Rashba coupling in a bulk metal that also globally preserves the inversion symmetry, although there is local inversion symmetry breaking by the buried interfaces. We have further demonstrated the crossover from EY-type to DP-type spin relaxation by tuning the interface density, which is usually difficult to achieve on the same material platform. We show a unique interplay of EPC and Rashba interaction that can lead to many-body quantum states like spin-orbital polarons (*53*).

## MATERIALS AND METHODS

### Material synthesis

Ag@Au NHs were synthesized via a two-step colloidal process (*23*, *24*). First, AgNO_3_ was reduced with ice-cold NaBH_4_ in ultrapure water (~18.2 M∙ohm·cm) containing NaOH, NH_4_Br, KI, and CTAB to form AgNPs. Then, HAuCl_4_ was added at 40°C under stirring to form a gold shell. The reaction was monitored by ultraviolet-visible spectroscopy and quenched with isopropyl alcohol (IPA) , followed by centrifugation at 10,000 rpm for 30 to 45 min to remove excess CTAB.

The NHs were drop-cast onto prepatterned Cr/Au (~10/70 nm) electrodes on glass substrate. Samples were dissolved in CHCl_3_, dried at 60°C, and washed sequentially with deionized water, KOH, and IPA to remove residual CTAB and promote sintering. This cycle was repeated 10 times to form films of thickness, *t* ≈ 3 ± 0.5 μm (see fig. S2).

### Electrical transport measurements

Four-probe resistivity measurements of the Ag@Au NHs were carried out in home-built cryostats down to ~6 K using a DC current of ~100 μA sourced by a Keithley 6221, while the voltage was measured using a Keithley 2182A nanovoltmeter. A Keithley 3700 multiplexer card enabled voltage acquisition across multiple contact pairs. Delta-mode voltage measurement was used to suppress thermal electromotive force (thermo-EMF) (see section S4.1). For lower temperatures (*T* ~ 10 to 0.3 K), resistivity and magnetotransport measurements were performed in a He-3 cryostat. The tunelling measurements were performed in another home-built cryostat that can cool down to 5 K and hosts a specialized tip-sample chamber with nanopositioners (attocubes and piezo tubes) connected to the tip holder and electrical contacts attached to both the tip and the film. A sharp Pt/Rh metallic tip is brought in contact with the film in a controlled manner with the help of the nanopositioners as indicated in the schematic of the experimental setup in Fig. 5A of main manuscript.

### Analysis of quantum transport data

Quantum transport data was analyzed using WL, WAL, and EEI corrections to extract phase coherence time ( $\tau_{\phi }$ ) and spin orbit scattering time ( $\tau_{\text{soc}}$ ). The Maekawa-Fukuyama model was applied for WL/WAL effects, while a 3D model was used for both WL/WAL and EEI due to the isotropic MR observed in the films. The conductivity corrections were then fitted to the experimental data. The total quantum correction to conductivity is shown in Eq. 1 of the main text. Here, ${\left.\frac{\Delta \rho }{\rho^{2}}\right|}_{\text{QI}}$ and ${\left.\frac{\Delta \rho }{\rho^{2}}\right|}_{\text{EEI}}$ are the conductivity corrections from quantum interference and EEI effects, respectively (*29*, *32*).

### Conductivity correction due to quantum interference

$$\begin{array}{c} {\left.\frac{\Delta \rho }{\rho^{2}}\right|}_{\text{QI}}=\frac{e^{2}}{2\pi^{2}\hbar }\sqrt{\frac{\mathrm{eB}}{\hbar }}\left\{\frac{1}{2\sqrt{1-\gamma }}\left[f_{3}\left(\frac{B}{B_{-}}\right)-f_{3}\left(\frac{B}{B_{+}}\right)\right]\right\}\\ -f_{3}\left(\frac{B}{B_{2}}\right)-\sqrt{\frac{4B_{\text{soc}}}{3B}}\left[\frac{1}{\sqrt{1-\gamma }}\left(\sqrt{t_{+}}-\sqrt{t_{-}}\right)+\left(\sqrt{t}+\sqrt{t+1}\right)\right] \end{array}$$ (2)

where

$$f_{3}\left(z\right)=\Sigma_{n=0}^{\infty }\left[2\left(\sqrt{n+1+z}-\sqrt{n+z}\right)-\frac{1}{n+\frac{1}{2}+z}\right]$$ (3)

Here, $B_{\phi }=\hbar /4\mathrm{eD}\tau_{\phi }$ and $B_{\text{soc}}=\hbar /4\mathrm{eD}\tau_{\text{soc}}$ are the scales of phase-breaking magnetic field and spin-orbit magnetic field, respectively. $t=3B_{\phi }/4B_{\text{soc}}$ qualitatively represents the strength of WL to WAL, $\gamma ={\left(3g^{*}\mu_{B}B/8{\mathrm{eDB}}_{\text{soc}}\right)}^{2}$ represents the strength of Zeeman splitting, where $g^{*}$ is the effective *g* factor. $B_{\pm },B_{2}$ are characteristic fields given as $B_{\pm }=B_{\phi }+\frac{2}{3}B_{\text{so}}\left(1\pm \sqrt{1-\gamma }\right)$ , and $B_{2}=B_{\phi }+\frac{4}{3}B_{\text{so}}$ . It is to be noted that we have not considered any spin-flip scattering that can arise from magnetic impurities, thus implicitly making the phase-coherent and inelastic scattering processes equivalent, i.e., $\tau_{i}=\tau_{\phi }$ , $\tau_{i}$ being the inelastic scattering time.

### Conductivity correction due to EEI

$$\begin{array}{c} {\left.\frac{\Delta \rho }{\rho^{2}}\right|}_{\text{EEI}}=\frac{8e^{2}}{3\pi^{2}\hbar }F_{\sigma }\left[{\left(1+\frac{F_{\sigma }}{2}\right)}^{3/2}-1-\frac{3F_{\sigma }}{4}\right]\sqrt{\frac{T}{2D}}g_{3}\left(h\right) \end{array}$$ (4)

where

$$F_{\sigma }=\frac{\int d\hat{\Omega }v\left(q=2k_{F}\text{sin}\left(\theta /2\right)\right)}{\int d\hat{\Omega }v\left(0\right)}$$ (5)

is the average of the Coulomb interaction *v*(*q*) on the Fermi surface over the solid angle $\hat{\Omega }$ (*34*).

## Supplementary Materials

### This PDF file includes:

Sections S1 to S9 Figs. S1 to S16
