# Supplementary material for: Emergent Rashba spin-orbit coupling in bulk gold with buried network of nanoscale interfaces
Source: Sci Adv. 2025 Oct 10;11(41):eadz1680. doi: 10.1126/sciadv.adz1680 (PMC12513416; doi:10.1126/sciadv.adz1680)
Supplement: Supplementary file 1 — Sections S1 to S9 Figs. S1 to S16 [file sciadv.adz1680_sm.pdf]

Science Advances
AAAS

Supplementary Materials for

Emergent Rashba spin-orbit coupling in bulk gold with buried network of nanoscale interfaces

Shreya Kumbhakar *et al.*

Corresponding author: Shreya Kumbhakar, shreyak@iisc.ac.in; Banashree Debnath, banashreed@iisc.ac.in; Arindam Ghosh, arindam@iisc.ac.in

Sci. Adv. **11**, eadz1680 (2025)
DOI: 10.1126/sciadv.adz1680

This PDF file includes:

Sections S1 to S9
Figs. S1 to S16

S1 Material synthesis

Synthesis of AgNP:

Silver nanoparticles (AgNPs) were prepared by reducing $AgNO_3$ (1 mM) using ice-cold $NaBH_4$ (0.1 M) as a reducing agent. CTAB (0.1 M) was added as a stabilizing agent to control the nanoparticle size and uniformity. The reaction mixture, which also contained NH_4Br (1 M), was maintained at 40°C, with its pH slightly adjusted to basic conditions using NaOH (0.1 M). To ensure a high-yield synthesis of uniform AgNPs, KI (0.1 M) was incorporated. Upon the introduction of $AgNO_3$, the solution turned whitish and turbid, signifying the formation of insoluble silver-halide (AgX) clusters. After a carefully monitored waiting period (*23*), $NaBH_4$ was gradually added, resulting in an instantaneous color shift to yellow, indicating AgNP formation. This was further verified by monitoring the surface plasmon resonance (SPR) of the colloidal solution, which has a distinct peak at 393 nm from AgNPs (*23, 24*). While preparing pure AgNP film, the reaction is terminated with isopropyl alcohol (IPA) after the formation of AgNPs. The solution was centrifuged at $10,000$ rpm for 60 minutes to precipitate the nanoparticles.

Synthesis of Ag@Au nanohybrid:

To fabricate the Ag@Au nanohybrids, $HAuCl_4$ (1 mM) was introduced into the AgNP solution, leading to a colour transformation from yellow to brown, confirming the formation of an Au shell around the Ag core. A red shift in the UV-Vis absorption spectrum further validated the formation of the Au shell and the formation of the Ag@Au nanohybrid (NH) structure (*23, 24*). The reaction was terminated by adding isopropyl alcohol (IPA), facilitating nanoparticle precipitation. The solution was then subjected to centrifugation at $10,000$ rpm for $30 - 45$ minutes, after which the supernatant was removed, and the nanohybrids were collected for further processing.

Synthesis of Au NP:

The Au nanoparticles (Au NPs) were synthesized following the same protocol as AgNPs, with $HAuCl_4$ replacing $AgNO_3$ while keeping the other experimental parameters unchanged. The reduction of $HAuCl_4$ with $NaBH_4$ (0.1 M) induced a reddish-purple colour shift, marking the successful formation of Au NPs. The reaction was terminated by the addition of isopropyl alcohol (IPA), and the nanoparticles were precipitated by centrifugation at $10,000$ rpm for 60 minutes. The obtained solid sample was then used for further processing (*23, 24*).

S2 Structural characterization

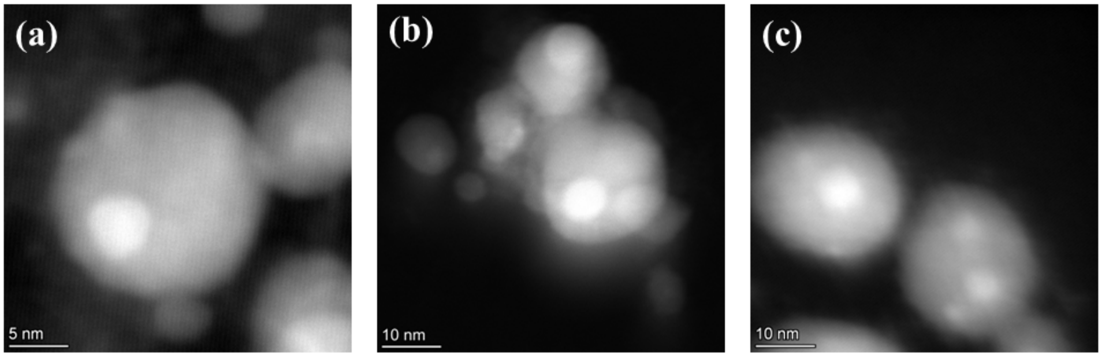

Figure S1: **HAADF imaging:** High-Angle Annular Dark-Field imaging (HAADF) images of Ag@Au nanohybrids are shown at various values of F: (a) F = 0.57,(b) F = 0.2, and (c) F = 0.13. The darker crystalline region of Au hosts a dispersion of the brighter region of Ag of diameter $\sim$ 2 nm. We clearly observe the integrity of the spherical AgNPs in the embedded structure at all F. We also note the gradual decrease of the average separation between the embedded interfaces with increasing F, again pointing towards robust structural integrity even with dense packing of interfaces.

S3 Film preparation

The Ag@Au NH films were prepared using a drop-casting method onto pre-patterned Cr/Au contacts (10 nm/70 nm) in a Van der Pauw or Hall bar configuration on a glass substrate. The substrate was prepared using optical photolithography, followed by metal deposition and a lift-off process to pattern the Cr/Au contacts. Before drop-casting, the Ag@Au NH solution was centrifuged, dried under vacuum ($\approx$1 mbar), redispersed in chloroform, and sonicated. The drop-casting process involved sequential drying at $50-60$°C, washing with DI water and KOH to remove CTAB, followed by rinsing in IPA (*23, 24*). This cycle was repeated $5-6$ times, resulting in films with an average thickness of $\approx 2-3\pm$ 0.5 µm over a 1 mm$\times$100 µm area, as shown in Fig S2. We have scanned the thickness profile across the black double arrow line as shown in the inset

of Fig S2. For the preparation of patterned nanoparticle films, Ag@Au NHs were drop-cast onto a patterned photoresist-coated substrate, followed by multiple cleaning cycles (5 – 6 times) as mentioned to remove excess residual chemicals. Finally, the polymer was removed using thermal treatment, leaving behind the patterned nanoparticle film on the Cr/Au leads. Finally, all films were coated with PMMA and sealed with a glass cover slip for encapsulation.

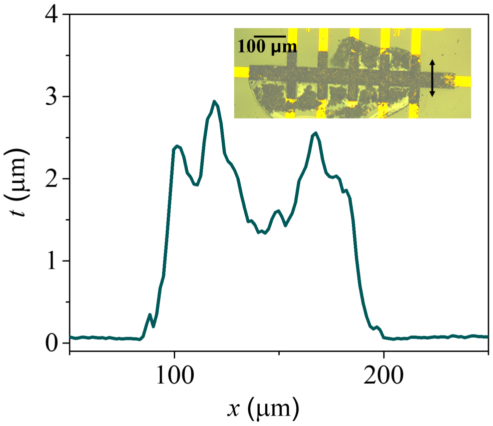

Figure S2: **Thickness measurement of a typical film:** Line scan showing the thickness (t) profile of a typical film, shown in the inset, is measured using optical profilometry along the double-arrowed line.

S4 Details of electrical transport measurements

S4.1 Electrical resistivity measurements

Four-probe electrical transport measurements were performed by applying a DC current of 100 μA using a Keithley 6221 source meter and measuring the voltage drop with a Keithley 2182A nano-voltmeter. A Keithley 3700 multiplexer card was used to record resistance across multiple channels simultaneously. Measurements were conducted in delta mode to minimize thermal electromotive force (EMF) errors, which cancel unwanted voltage offsets by alternating the current direction. For measuring the temperature dependence of the resistivity from 300 K to low temperatures. The experiment was carried out in a home-built variable temperature cryostat, capable of cooling the sample down to $\approx$ 6 K.

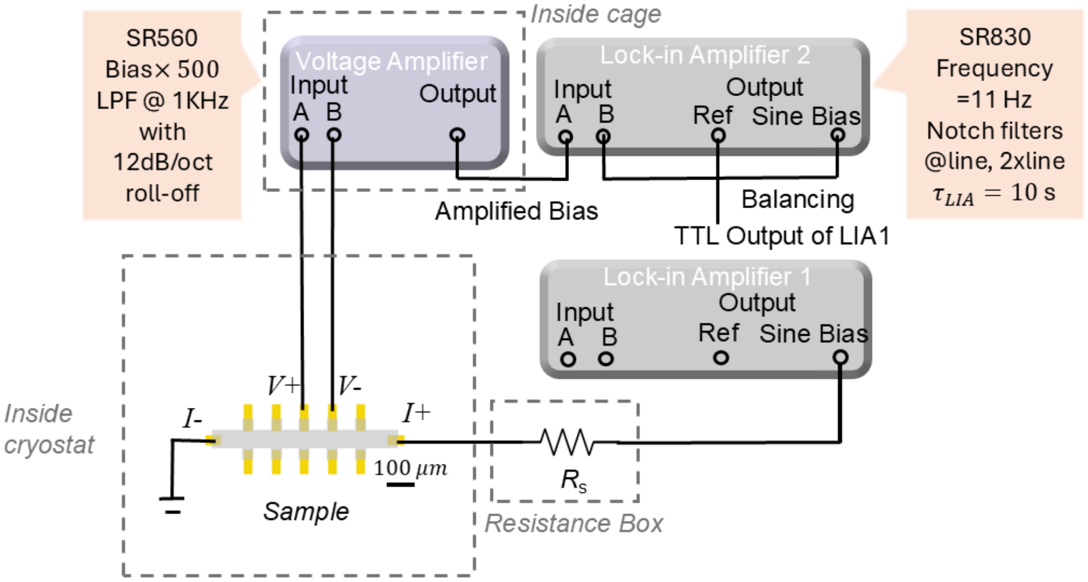

Figure S3: **Schematic of experimental set-up for magnetotransport measurements.** A four-probe measurement technique has been implemented to measure the magnetoresistance in the films. The shaded grey rectangular region indicates the sample prepared on the Cr/Au contacts, indicated by the gold-colored regions. The voltage signal from the sample has been amplified using a voltage amplifier (SR560), indicated by the purple box and then subsequently read out using a lock-in amplifier (SR830), indicated by the grey box, after balancing. The parameters used for the amplifiers have been mentioned in the schematic and further elaborated in the text.

S4.2 Magnetotransport measurements

Magnetotransport measurements were conducted in a commercial He-3 cryostat from Janis systems with magnetic fields up to ±5 T and temperatures ranging from 0.28 K−30 K. An AC measurement technique was implemented where an SR830 lock-in amplifier was used to drive current through sample contact $I+$ at an excitation frequency of 11 Hz. Notch filters were implemented to eliminate noise from line frequency (∼ 50 Hz) and its second harmonic. At the same time, the voltage signal was amplified using an SR560 low-noise voltage amplifier with a gain factor of 500. A 1 kHz low-pass filter (12 dB/octave roll-off) was implemented to improve the signal-to-noise ratio. The measurement schematic is shown in Fig. S3. The amplifiers were placed inside a Faraday cage for electromagnetic shielding. The Faraday cage is constructed of adjacent aluminium and mild

steel sheets, both having a thickness of 1.5 mm. The shielding effectiveness is determined by the skin depth (δ) of the particular metal at different frequencies. The thickness of the shield is typically kept as $3-4$ times δ, ensuring effective shielding, where δ=$\sqrt{1/\pi\sigma\mu f}$ (f is the frequency, $\mu = \mu_0\mu_r$ is the permeability, and σ is the conductivity of the metal). Using this, one obtains $\delta_{\mathrm{Al}} \approx 0.83$ mm at 10 kHz and $\delta_{\mathrm{steel}} \approx 0.20$ mm at 1 kHz. Hence, the Faraday cage effectively screens out high-frequency noise.

For performing magnetotransport measurements, a strategy of *hold and ramp* has been implemented to record the data. Specifically, the magnet is first ramped to the desired magnetic field, and the persistent heater is switched off. The data was recorded after a particular wait time (determined by the data acquisition rate) of *holding* the magnet in persistent mode, ensuring a steady magnetic field and avoiding the possibility of inducing eddy currents. A lock-in time constant of 10 s is used to average the signal, effectively filtering out high-frequency noise and improving the signal-to-noise ratio. The persistent switch heater is then switched on again, and the magnet is *ramped* to the next desired field.

S4.3 Hall measurements

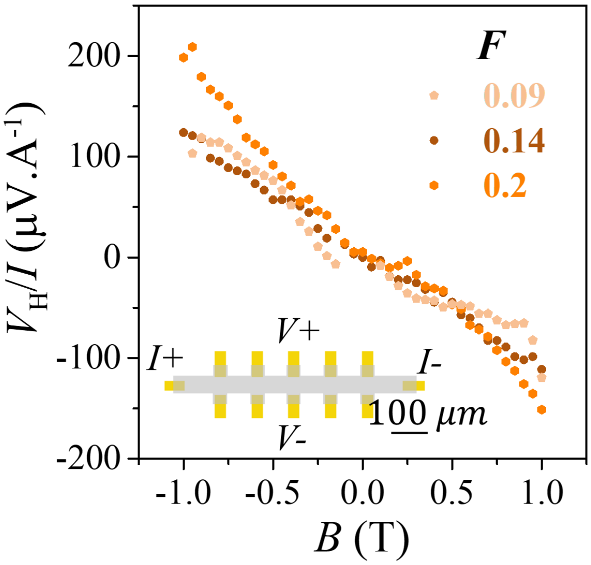

Figure S4: **Hall measurements:** Hall measurements at $T \sim 0.3$ K are shown for films with varying Ag volume fraction, $F = V_{Ag}/(V_{Ag} + V_{Au})$. The inset shows the schematic of lead geometry used for Hall measurements. Yellow lines show the deposited Cr/Au electrodes, and the light grey region is the dropcast film. $I+, I-$ and $V+, V-$ indicate the current and voltage probes respectively. The electron density is estimated to be n ($\sim 10^{28}$ m^{-3}) for all F-values from the measured Hall resistance ($V_H/I = 1/ne$, V_H, and I are the measured hall voltage and current, respectively). .

S5 Evidence of quantum transport

Quantum transport refers to the manifestation of quantum interference effects in the electrical transport properties. Briefly, in disordered conductors, electrons can travel in self-intersecting loops due to enhanced momentum scattering by disorder. At low values of T, when the inelastic scattering rate or the dephasing rate of the electrons reduces, the electrons in the forward and backwards-travelling paths can interfere constructively or destructively, giving rise to WL or WAL, respectively. In materials with strong spin-orbit coupling, WAL dominates at low magnetic fields, while WL emerges at higher fields. Both temperature dependence and angle dependence are powerful tools to confirm the quantum nature of WL/WAL effects. Our temperature-dependent measurements, in Fig. 2(A) of main manuscript and Fig. S5, reveal that the WL contribution weakens with increasing temperature, consistent with reduced τ_ϕ with decreasing T, observed in diverse materials, while the WAL component remains largely unaffected. Also, we note that at higher values of F, there is an enhancement of MR at higher magnetic fields. We attribute this to the electron-electron interaction effects (EEI), which are in competition with the quantum interference effects. It should be noted that the quantum interference effects *i.e.* WL/WAL and the EEI effects differ in T and B dependences [See Methods of the main manuscript]. While quantum interference gets suppressed with increasing T (Fig. 2(A) and Fig. S5) as the dephasing rate of the electron increases, the magnetic field-dependent component of EEI enhances with T and always exhibits positive MR. Thus, the magnitude of MR alone is not a direct measure of WL/WAL. To make this point clearer, we present below in Fig. S5 the temperature dependence of MR up to $T \sim 30$ K for a sample with $F = 0.09$. Since EEI, which is not a quantum interference effect, is minimal here, the suppression of WL/WAL effects is clearer in this sample.

S5.1 Temperature dependence of MR

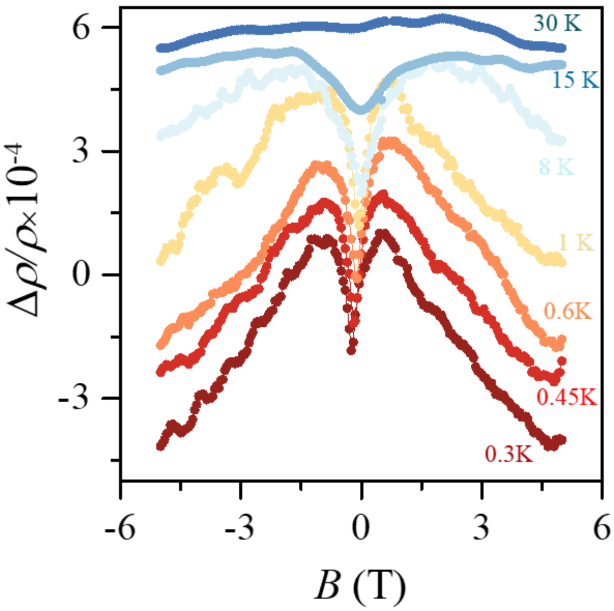

Figure S5: **Temperature-dependent magnetoresistance measurements:** Temperature dependence of MR of a film with Ag-filling fraction, $F = 0.09$, from $T \sim 0.3$ K to 30 K. We clearly observe the suppression of MR with increasing T.

S5.2 Angle dependence of MR

Angle-dependent MR measurements in Fig. S6 show that the MR remains nearly unchanged between θ= 0°(magnetic field parallel to the sample plane) and 90°(magnetic field perpendicular to the sample plane) for all temperatures, indicating isotropic behavior. This is not unexpected because of the three-dimensional nature of our film, where the thickness of the film exceeds the phase-breaking and other length scales by two to three orders of magnitude. This justifies the use of a 3D theoretical model, as discussed in the Methods of the main manuscript.

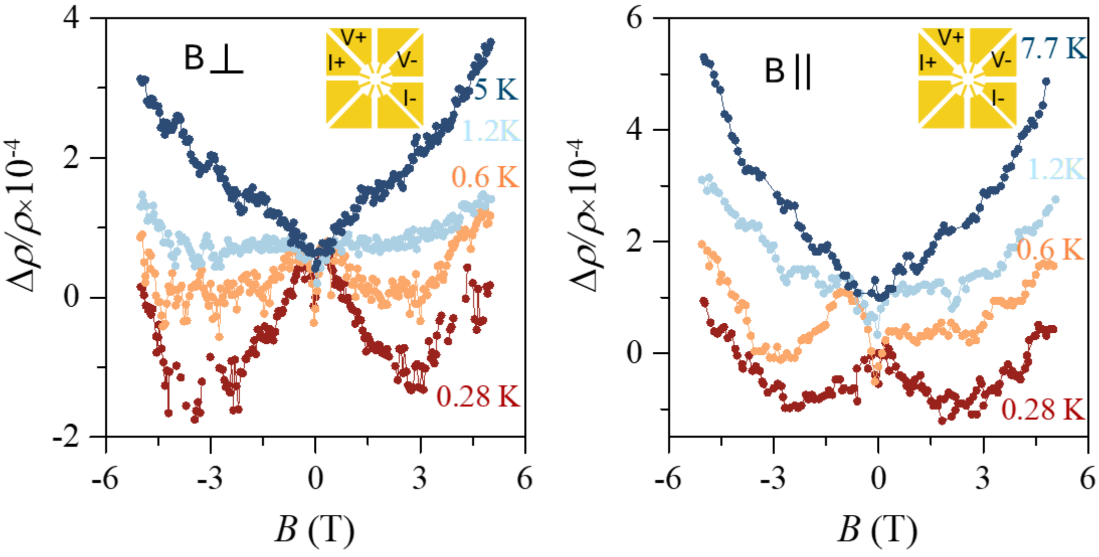

Figure S6: **Angle-dependent magnetoresistance measurements:**Angle-dependent MR of F = 0.14 showing isotropic behavior at T = 0.3 K.

S6 Analysis of the fitting parameters of the magnetoresistance data

$H_\phi(l_\phi), H_{\mathrm{soc}}(l_{\mathrm{soc}}), \mathcal{F}_\sigma$, and g are the parameters obtained by fitting the magnetoresistance data shown in Fig. S7 using Eq. [1] of the main manuscript.

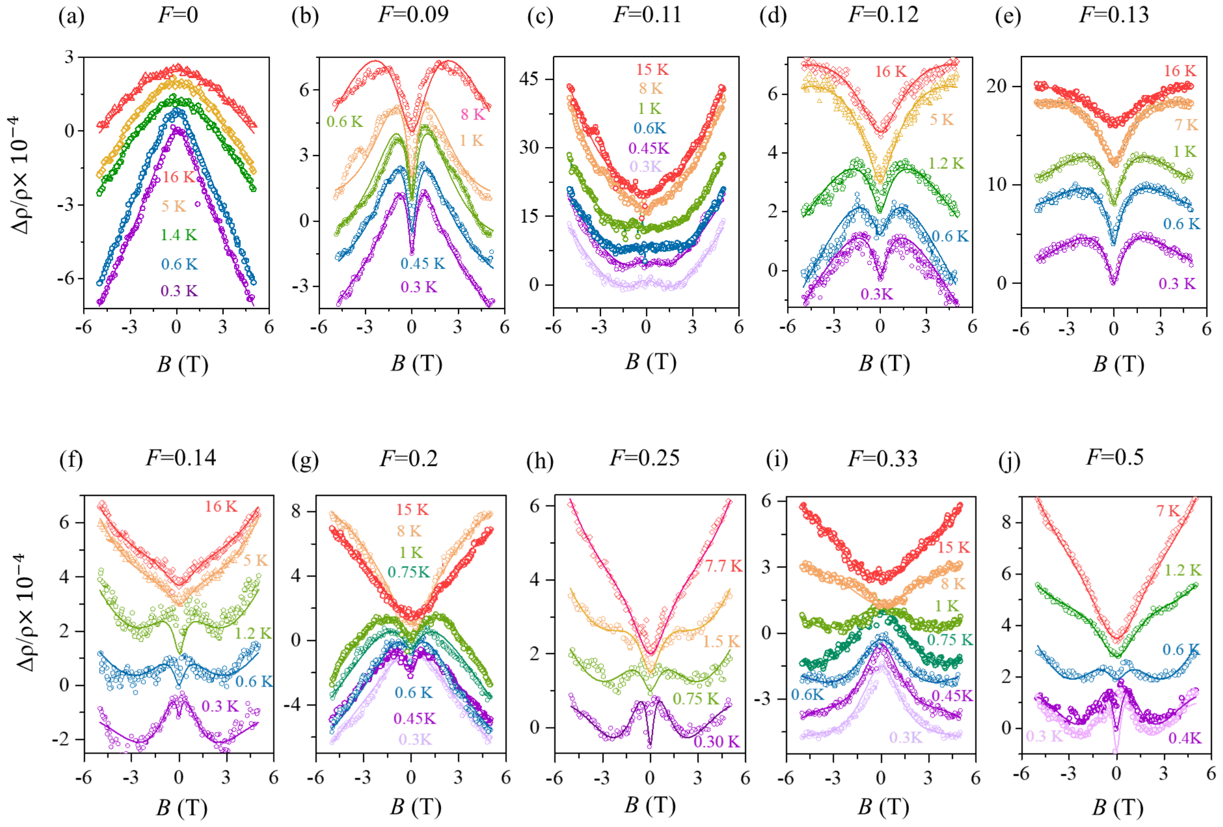

Figure S7: **Fitting of magnetoresistance data:** Magnetoresistance (MR), represented by the relative change in resistivity $((\rho(B) - \rho(0))/\rho = \Delta\rho/\rho)$ with magnetic field, in a perpendicular magnetic field is measured for films with varying Ag/Au interface density, $F = 0, 0.09, 0.11, 0.12, 0.13, 0.14, 0.2, 0.25, 0.33, 0.5$. $F = 0$ represents pure AuNP film. Solid lines represent fits to the data using conductivity corrections from quantum interference and electron-electron interaction effects (Eq. [1] of the main manuscript). The data in all panels have been shifted vertically for visual clarity.

S6.1 Phase-coherent and spin-orbit scattering rates

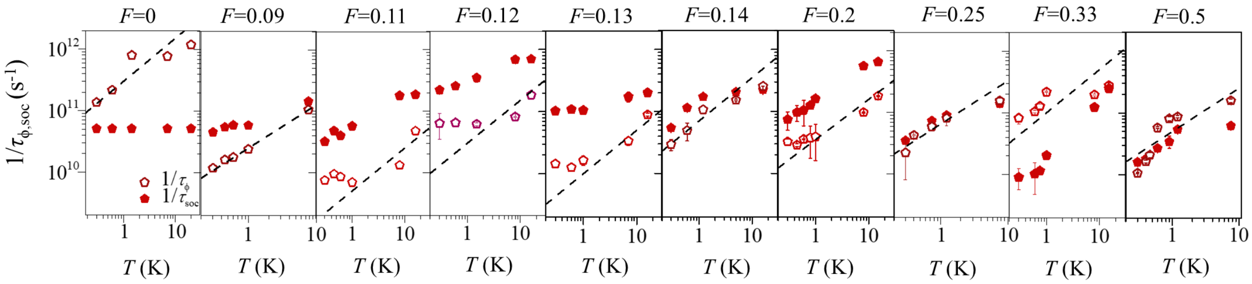

Figure S8: **Phase-coherent and spin-orbit scattering rates:**Temperature (T)-dependences of spin-orbit interaction rate ($1/\tau_{\mathrm{soc}}$) and phase-breaking rate ($1/\tau_\phi$) are shown for varying values of F. The dotted lines in all panels represent $1/\tau_\phi^{-p}$ with $p \sim 0.7 - 1$.

S6.2 Phase breaking length

The phase coherence length l_ϕ is estimated from the phase breaking magnetic field H_ϕ as $l_\phi = \sqrt{\hbar/4eH_\phi}$. For all values of F, l_ϕ ranges within $10 - 100$ nm with a consistent decrease towards higher-F, indicating a higher phase-breaking rate.

S6.3 Average screened Coulomb potential, $\mathcal{F}_\sigma$

S6.4 g-values

The effective g-factor in a system is such that the energy between the Zeeman split spin states is $g_{\mathrm{eff}} = mu_B B$, B being the applied magnetic field. For a free electron, $g = 2$. However, in a solid state system, this number can be drastically different. For example, if there is no orbital moment or if it is quenched, the spin-orbit interaction mixes the spin-up and spin-down states, thus reducing the g-factor (*39*). In the presence of orbital contributions, SOC increases the electron moment, thus increasing the values of g (*54*). Additional factors contribute in low-dimensional systems as compared to bulk systems. For example in metal nanoparticles, interface scattering reduces the orbital contributions, thereby decreasing g (*55–57*). Such quantum confinement effects also give rise to mesoscopic fluctuations in the values of g (*58*). It was observed that in Au nanoparticles

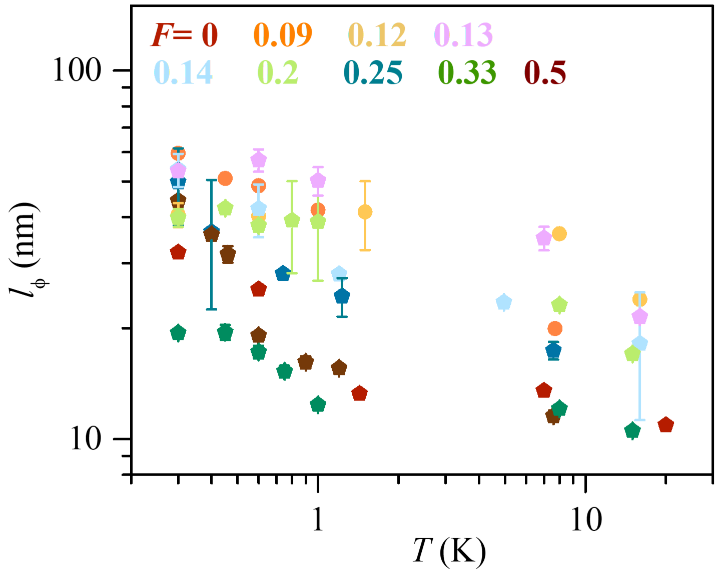

Figure S9: **Phase breaking length:** Temperature (T) dependence of the phase coherence length l_ϕ is shown for different values of F.

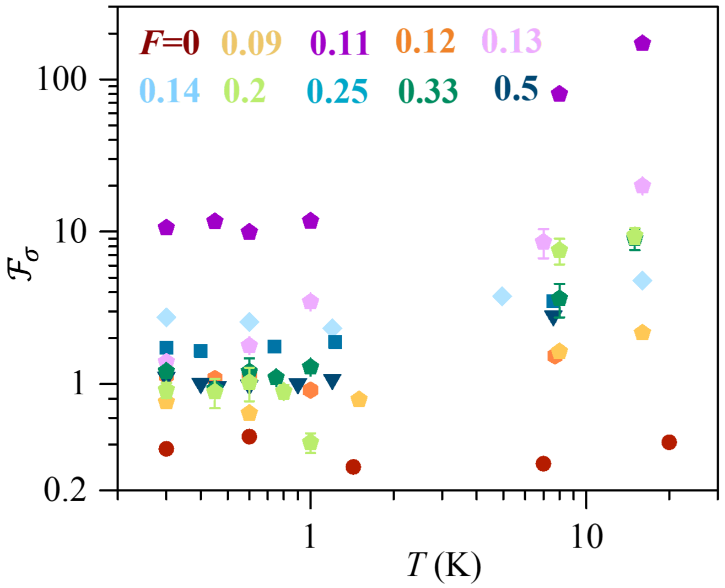

Figure S10: **Coulomb Screening Parameter:** $\mathcal{F}_\sigma$, computed from fits to the MR data, is shown for different films with varying F.

the g-factor is reduced from the free electron value of 2 to about 0.3 (*56, 57*). All the discussions above considered electron-electron interactions. In the absence of orbital contribution, the effect of EEI and SOC are competing in nature as the former tends to lift the spin degeneracy via exchange

interactions thus increasing net spin of a many-electron state and hence the effective g-value whereas the latter mixes the spin states thus decreasing g (*59*).

In our experiments, we observe g for Au nanoparticle film to be ~ 0.2, consistent with previous studies on Au nanoparticles (*56, 57*). With finite Ag filling at $F = 0.09$, the g-factor increases and has an increasing T-dependence as well. Interestingly, we also observe g to increase till $F \sim 0.14$, and it decreases again for $F = 0.5$. This resembles the dependence of $\tau_{\rm soc}$ on F and is consistent with a spin-orbit coupled state where the orbital contribution and strong SOC increases values of g (*54*).

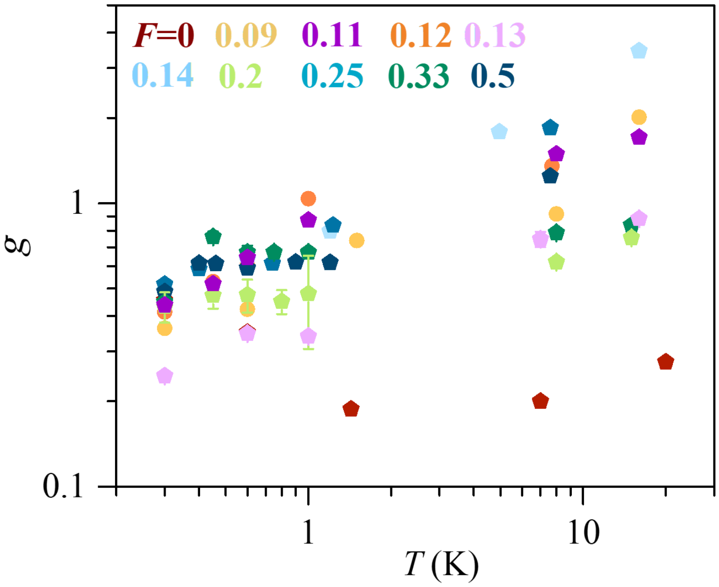

Figure S11: **Lande g-factor:** Temperature (T)-dependence of the effective electron Lande g-factor is shown for films with varying F.

S7 Details of theoretical computation

To estimate the charge transfer at the Ag/Au interfaces due to the difference of onsite electrochemical potentials, the electronic structure was modeled using a semi-phenomenological tight-binding model constructed from the s-like conduction bands of Au and Ag. The model includes uniform nearest neighbor hopping, site energy offsets to account for the work function mismatch between the two elements, and both on-site and long-range Coulomb interactions. These interactions were

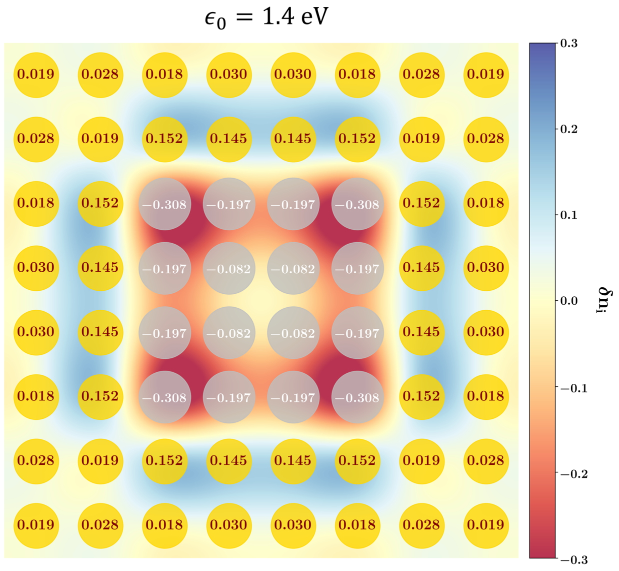

Figure S12: **Theoretical estimation of the charge distribution in a model system of Ag atoms surrounded by Au atoms.** Excess electron occupancy $\delta\mathbf{n}_\mathrm{i}$ at each atomic site i, of a 2D square lattice with Ag embedded within a matrix of Au atoms.

treated within the Hartree approximation, enabling a self-consistent determination of the Hartree potentials and the resulting charge redistribution across the lattice sites. To facilitate analytical exploration and for the ease of studying the effects of parameter variation, we studied a simplified two-dimensional square superlattice, consisting of periodic (4×4) Ag clusters embedded within an Au matrix, forming a 64-site unit cell. This toy model captures the essential physics of interface-induced charge inhomogeneity and enables controlled studies of electron-electron and electron-phonon coupling effects. The charge transfer in this lattice for an onsite electrochemical potential difference, $\epsilon_0 = 1.4$ eV, is shown in Fig. S12. From the excess electron occupancy ($\delta\mathbf{r}_\mathrm{i}$) profile, we note electron doping from Ag to Au, as depicted schematically in Fig. 4(E) of the main manuscript. For more details on the theoretical calculations, please refer to (*25*).

S8 Tunelling measurements

The tunelling measurements were performed by bringing a sharp Pt/Rh metallic tip close to the film in a controlled manner with the help of nanopositioners (attocubes and piezoceramic cylinders)

as indicated in the schematic of the experimental set-up in Fig. 5(A) of the main manuscript. The tip contacts the sample with an effective diameter d. The tip-sample chamber is loaded inside a home-built cryostat that could be cooled down to $T \sim 5$ K. We have measured the differential resistance/conductance across the tip-sample contact in a four-probe configuration with the modulation spectroscopy technique (*24,52*). Specifically, a mixed AC+DC current, $I + \delta I \cos(\omega t)$, where I is the DC current, δI is the AC current and $\omega \sim 227$ Hz is the AC excitation frequency is sent to the tip while the sample is grounded. The AC bias, δV across the tip-sample contact is measured in a four-probe configuration at ω frequency and 0° phase under the corresponding DC biasing, giving the differential resistance as $R_t = \delta V/\delta I$. The DC current is varied to tune the DC bias (V) across the tip-sample contact in the desired energy (eV) range. A typical spectrum measured for film $F = 0.14$ at $T \sim 8$ K is shown in Fig. S13(a). Fig. S13(b) shows the tunelling

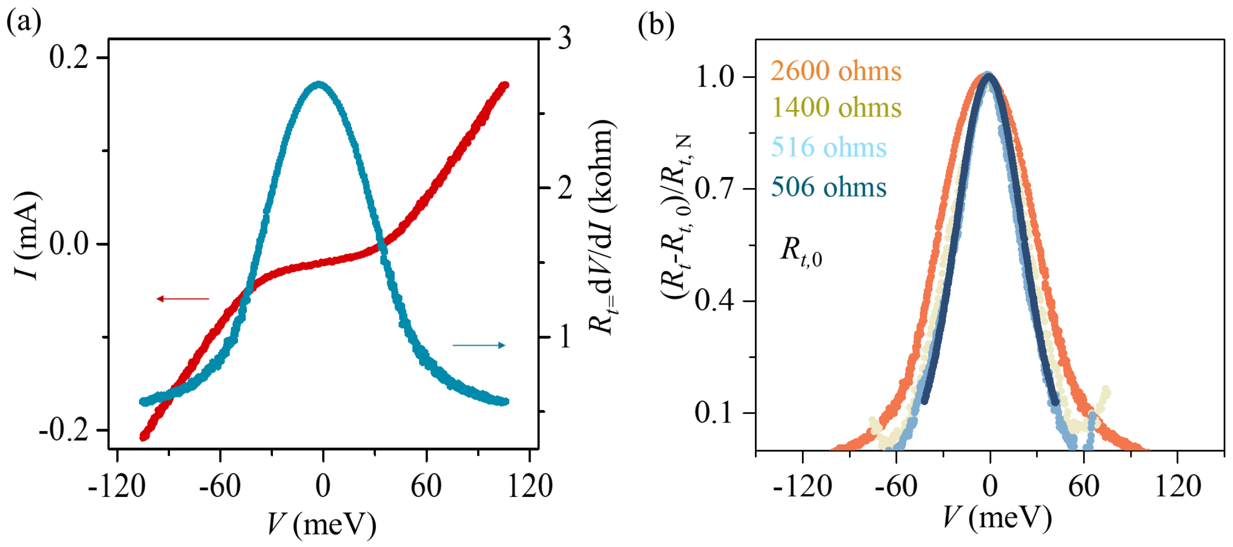

Figure S13: **Low-temperature electrical tunelling measurements in a typical film of the Ag@Au nanohybrid.** (a) The tunelling $I-V$ characteristics and the corresponding bias dependence of measured tunelling resistance, $R_t = \mathrm{d}V/\mathrm{d}I$ are shown for a typical film with Ag volume fraction $F = 0.14$ at $T \sim 8$ K. (b) shows the bias dependence of normalized R_t *i.e.* $(R_t - R_{t,N})/R_{t,0}$ measured at distinct positions of the sample, that collapse on each other.

spectra (R_t vs V) measured at different positions in the sample. R_t is normalized such that the resistance maximum ($R_{t,0}$) at zero bias is 1 and the value ($R_{t,N}$) saturating at higher bias is 0. The

collapse of the gap measured at different positions excludes any disorder-mediated scattering. R_t is inverted to obtain the tunelling conductance as $G_t = 1/R_t$.

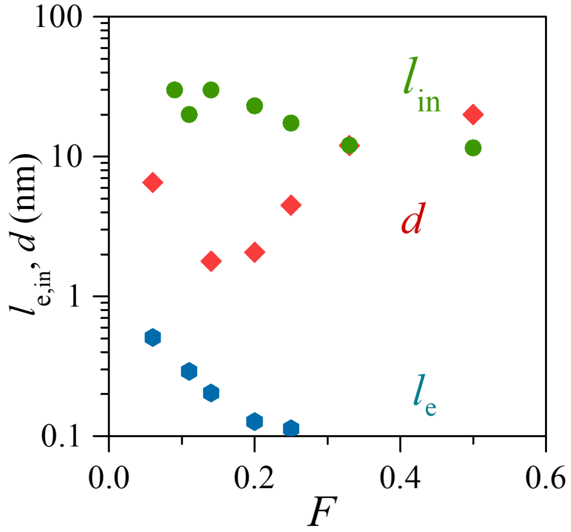

Figure S14: **Comparison of elastic (l_{el}), inelastic (l_{in}) length scales, and the tip-sample contact diameter (d) for different values of Ag-filling, F at temperature $T \sim 8$ K.** For $F \gtrsim 0.3, l_{el}$ becomes less than the interatomic spacing, indicating the inapplicability of the Drude expression at these resistivities to estimate the elastic scattering length.

Fig. S14 shows a comparison between the elastic (l_{el}), inelastic (l_{in}) length scales with the tip-sample contact diameter, d at different values. d is estimated from a diffusive transport regime as ρ/R_t. l_{in} is equivalent to the phase-breaking length (l_ϕ) that has been derived from the quantum transport measurements. We note that this equivalence holds under the assumption that there are no magnetic impurities in the system. $l_{el} = \sqrt{D\tau}$, where the diffusivity D and scattering time τ are estimated from the residual resistivity via Drude expression as discussed in the main text. We observe $d < l_{in}$, indicating that the collisions at the tip-sample contact are energy-conserving. Hence, the conductance directly maps the tunelling density of states, as illustrated schematically in the inset of Fig. 5(C) of the main manuscript.

Fig. S15 shows the T-dependence of the tunelling spectra. The behaviour changes to that of a metallic differential resistance. This can be attributed to increased inelastic collisions and resultant energy-relaxing processes at the tip-sample contact, due to which the tunelling DOS cannot be

probed. The T-dependence of the spectra enables us to exclude any defect-mediated tunnelling process as the origin of the dip in tunelling conductance.

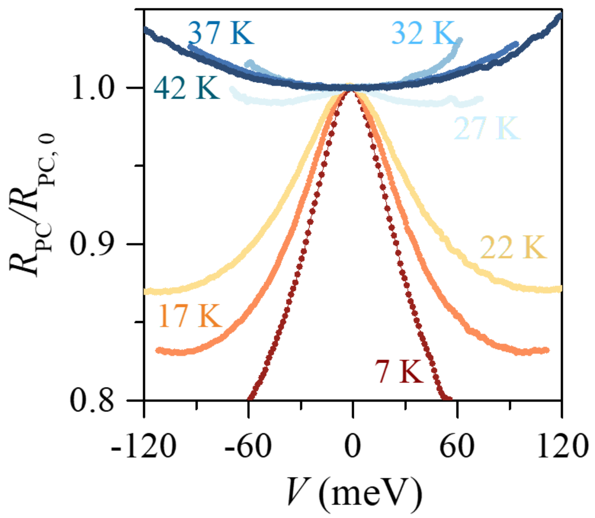

Figure S15: **Temperature-dependent electrical tunnelling measurements.** Tunnelling resistance (R_t) is measured in a film with F = 0.25 from temperature T ~ 7 K to 42 K. R_t is normalized by the respective zero bias resistance, $R_{t,0}$.

S9 Discussion on the crossover of spin-relaxation mechanism

It will be interesting to see if the crossover between the spin-relaxation mechanisms is reflected in the behaviour of the separation between interfaces, the Coulomb screening length, and the spatial extent of the confining potential at the interfaces. However, to rigorously quantify the latter two parameters, we will likely need ab-initio calculations of the structure, which are beyond the scope of the manuscript, as these crucially depend on the band structure, band hybridisation, electric permittivity, effective mass and so on. However, below we discuss some possible estimates of these quantities.

- The inter-particle spacing can be approximated by the expression $F/r_{\mathrm{Ag}}^{1/3}$, assuming a periodic distribution of nanoparticles. This is supported by our earlier paper (*23*), showing the linearity of the measured resistivity with the approximated area of buried interfaces per unit volume based on this expression.

- The Coulomb screening length ($l_{\mathrm{screening}}$) for bulk gold is 0.05 nm. In the Thomas-Fermi approximation of electrostatic screening, this is determined by the permittivity and the charge carrier density. From our low-field Hall measurements, shown in Supplementary Fig. S4, we do not yet see any appreciable change in the carrier density. However, the permittivity can widely vary with F. Indeed, our magnetotransport and tunnelling measurements indicate that the screening, captured by $\mathcal{F}_{\sigma}$, becomes poorer with increasing F, becoming the weakest at $F \sim 0.12$. We believe that at this value of F, where we see the crossover of spin-relaxation mechanism, and rather in a wide region spanning it, $l_{\mathrm{screening}}$ can far exceed the interparticle separation. Hence, to make a meaningful interpretation, we probably need to look at the spatial extent of the confining potential at the interface.

- The confining potential length at the Ag/Au interface can be approximated by the polaron delocalization length, which can widely vary depending on the specific material properties. For example, this typically ranges between 0.5-1 nm for small polarons in oxide perovskites (*60*), $1-5$ nm for small/large (mixed) polarons in TMDCs (*61*), $1-10$ nm for intermediate/large polarons in organic semiconductors (*61*), $4-10$ nm for large polarons in halide perovskites (*62*), $3-15$ nm for large polarons in conjugated polymers/crystals (*63,64*),

10-100 nm for large/delocalized polarons in inorganic semiconductors (*64*) and so on. Our temperature-dependent resistivity measurements (*24*) have shown that the electrical transport in the Ag@Au system deviates from a conventional metal and can be phenomenologically modelled by a thermally activated parallel channel. This has led us to believe that the Ag/Au interface likely hosts small polarons, which possess a thermally activated hopping mobility. By comparing the polaron delocalization length for small polarons in diverse systems, we assume the same in our system to be around 1 nm, which is an estimate of the confining potential or charge accumulation at the interface.

Below in Fig. S16, we plot the average separation between the adjacent interfaces as a function of F. The black solid line represents twice the confining potential depth. The intersection of the curves indicates a region where the conduction electrons of gold are the most likely to experience the surface potential from surrounding interfaces. Hence, within the statistical uncertainty of the size and spatial distribution of the silver nanoparticle, we observe that the crossover of the spin-relaxation mechanisms may correspond to the scale of the interface separation becoming of the order of the localization length at the interface.

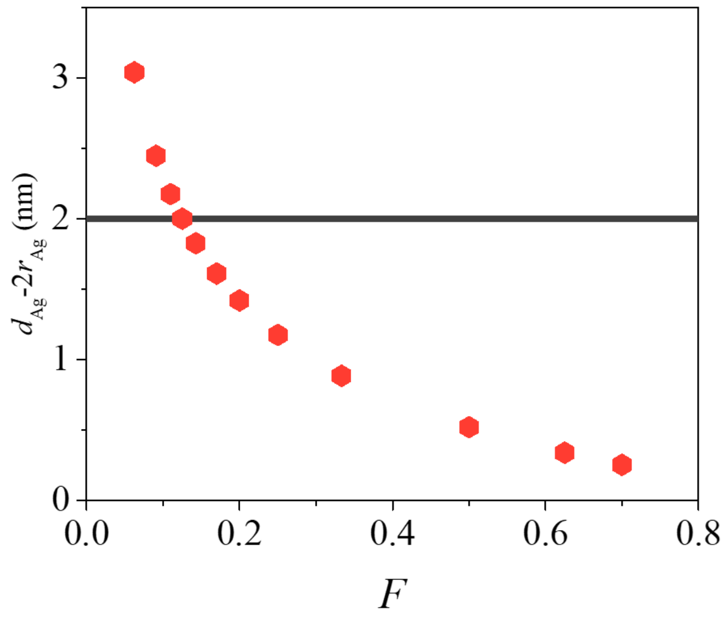

Figure S16: **Theoretical comparison between the average separation of the Ag nanoparticles embedded in Au and the confining potential depth at the interfaces of Ag and Au.** Average separation between adjacent interfaces plotted as a function of F, illustrating the variation in interfacial spacing with changing F. The black solid line indicates twice the confining potential depth at the interfaces.
